# Structural transition and magnetic properties of Mn doped Bi_0.88_Sm_0.12_FeO_3_ ceramics†

**DOI:** 10.1039/d0ra01642j

**Published:** 2020-03-26

**Authors:** N. T. Hien, N. D. Vinh, N. V. Dang, T. T. Trang, H. T. Van, T. T. Thao, L. T. Hue, P. T. Tho

**Affiliations:** Ceramics and Biomaterials Research Group, Advanced Institute of Materials Science, Ton Duc Thang University Ho Chi Minh City Vietnam nguyenthihien@tdtu.edu.vn; Faculty of Applied Sciences, Ton Duc Thang University Ho Chi Minh City Vietnam; Department of Physics and Chemistry, Thai Nguyen University of Sciences Thai Nguyen Vietnam thopt@tnus.edu.vn; Institute of Research and Development, Duy Tan University Da Nang 550000 Vietnam

## Abstract

We investigated the effects of Mn doping on the crystal structure, phonon vibration, and magnetic properties of Bi_0.88_Sm_0.12_FeO_3_ ceramics. Mn doping effectively modified the rhombohedral symmetry and induced a structural transition from an *R*3*c* rhombohedral to *Pnam* orthorhombic structure. Magnetic measurements revealed a weak ferromagnetic behavior, which was related to the canted antiferromagnetic order of the *Pnam* structure. The cycloidal spin structure of the *R*3*c* phase could not be suppressed by substitution of Mn at the Fe site. Studies on the self-phase transition and electric field-induced structural transition revealed many changes in coercivity and remanent magnetization, which are believed to originate from the *R*3*c*/*Pnam* phase switching along with spin frustration. Observations of the field step-dependent hysteresis loop and the ferromagnetic-like hysteresis loop after poling in an electric field provided direct evidence of phase boundary (PB) ferromagnetism and magnetic coupling at the PB.

## Abstract 2

We investigated the effects of Mn doping on the crystal structure, phonon vibration, and magnetic properties of Bi_0.88_Sm_0.12_FeO_3_ ceramics.

## Graphical abstract

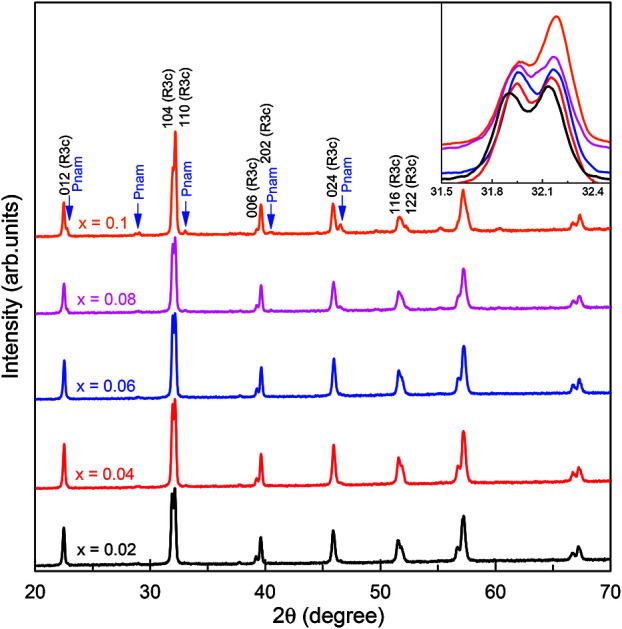

## 1. Introduction

The coexistence of energetically equivalent phases has recently received much interest in relation to BiFeO_3_-based multiferroics.^1–3^ The coexistence of phases with differences in lattice strain, magnetic anisotropy, and antiferroelectric orderings often offers extraordinary properties such as high electromechanical response, double and pinched ferroelectric hysteresis loops,^4,5^ vertical magnetic hysteresis loop shift (named as the exchange bias effect in some articles),^6–8^ and the self-change of magnetization with time.^9^ The enhancement of electromechanical responses, including the piezoelectric coefficient and dielectric constant, is believed to originate from the field-driven reversible phase transformation or from the monoclinic phase that allows the rotation of the ferroelectric polarization vector between two structures.^1,10,11^ Several reports have also stated that the incommensurate phase, a bridging phase between the rhombohedral and orthorhombic structures, can enhance the ferroelectric properties of rare earth-doped BiFeO_3_ (BFO).^12,13^ The double and pinched ferroelectric loops are attributed to the domain wall pinning, field-induced phase transition in the vicinity of a morphotropic phase boundary (MPB) and the coexistence of both the ferroelectric and antiferroelectric orders of the bridging phase.^4,14,15^ The vertical hysteresis shift (VHS) possibly originates from the minor loop effect. However, a remarkable enhancement of the VHS in MPB systems and in the nanocomposites is definitely abnormal.^7,8,16–18^ We observed that the VHS is strongly dependent on the phase ratio of two structures at the MPB.^7^ Therefore, an increase in the VHS at the MPB possibly occurs from magnetic coupling at the boundary or from the inhomogeneous magnetic anisotropy of the coexisting phase.^6,7,19^ The self-change of magnetization with time has been previously observed in (La, Co),^19^ (La, Ti),^9^ and (La, Zn) codoped BFO at the MPB.^17^ This effect is attributed to the isothermal structural transition along with spin frustration at the PB, which is an implication for the appearance of a PB ferromagnetic order embedded in the antiferromagnetic matrix.^9,19^ Magnetic coupling between two structural phases and the PB is similar to the interaction of the antiferromagnetic–ferromagnetic multilayer.^20^ PB ferromagnetism and magnetic exchange anisotropy were used to explain the VHS, classical exchange bias effect, and field step-dependent hysteresis loop.^6,7,9,19^ Moreover, the enhancement of magnetization at the MPB of BFO-based compounds can also be fulfilled by considering the contribution of PB ferromagnetism.^13,21–23^ To date, numerous attempts have been made to study the effects of Sm and Mn substitution on the crystal structure and on the ferroelectric and magnetic properties of BFO multiferroic.^24–28^ However, these reports do not provide a clear understanding of the correlation between the coexistence phase and the ferroelectric/ferromagnetic properties of Mn doping on Bi_1−*y*_Sm_*y*_FeO_3_ compounds. For instance, Zhou *et al.* reported the crystal structure and multiferroic properties of Bi_1−*y*_Sm_*y*_Fe_0.95_Mn_0.05_O_3_ (*y* = 0.08 and 0.16) ceramics.^27^ Pandey *et al.* studied the structural evolution of Bi_1−*y*_Sm_*y*_Fe_1−*y*_Mn_*y*_O_3_ (0 ≤ *y* ≤ 0.15) compounds and observed maximum magnetization at the PB, where the percentage of two phases has a comparable value.^25^ Therefore, these investigations do not answer the question of how Mn can induce structure transition and whether the existence of two structural phases can enhance ferroelectric/ferromagnetic properties in the vicinity of an MPB. It is accepted that Bi_1−*y*_Sm_*y*_FeO_3_ mother compounds show coexistence of the *R*3*c* rhombohedral and PbZrO_3_-type orthorhombic structures in the composition range of 0.1 ≤ *y* < 0.14.^29,30^ Therefore, in the present paper, we investigate the crystal structure and magnetic properties of Bi_0.88_Sm_0.12_Fe_1−*x*_Mn_*x*_O_3_ (0.02 ≤ *x* ≤ 0.1) compounds at the polymorphs of the polar *R*3*c* rhombohedral and antipolar *Pnam* orthorhombic structures. The analysis of X-ray diffraction (XRD) patterns and Raman spectra revealed that Mn doping induces structure distortion and structural transition. By varying magnetization with time, the present study showed an enhancement of coercivity and a reduction of magnetization. The VHS was observed in all the samples, and its value tended to increase with a decrease in the *R*3*c*/*Pnam* phase ratio. In particular, through the electric field-induced structural transition, we showed evidence of contribution of PB ferromagnetism to the magnetic properties of Bi_0.88_Sm_0.12_Fe_1−*x*_Mn_*x*_O_3_ ceramic compounds.

## 2. Experimental details

Polycrystalline compounds of the form Bi_0.88_Sm_0.12_Fe_1−*x*_Mn_*x*_O_3_ (BSFMO) with *x* = 0.02–0.1 were prepared through a conventional solid-state reaction. High-purity Bi_2_O_3_, Sm_2_O_3_, Fe_2_O_3_, and MnO_2_ powders were used as precursors. These powders with a specific composition were mixed, carefully ground, and pre-annealed at 900 °C in air for 24 h. The pre-annealed samples were re-ground, pressed into pellets, and finally sintered in air at 930 °C for 12 h. The crystalline structure and phonon characteristics of the fabricated samples were studied using an X-ray diffractometer (Miniflex Rigaku) equipped with a Cu-Kα radiation source (*λ* = 1.5405 Å) and by Raman scattering spectroscopy (LabRAM HR Evolution, Horiba) with an excitation wavelength of *λ* = 532 nm. The morphology and composition of the samples were examined by scanning electron microscopy (Hitachi S-4800). The XRD data were analyzed by the Rietveld method using the GSAS-2 program. Magnetization measurements were performed on a VSM LakeShore 7400. All investigations were carried out at room temperature.

## 3. Results and discussion

**Fig. 1:**
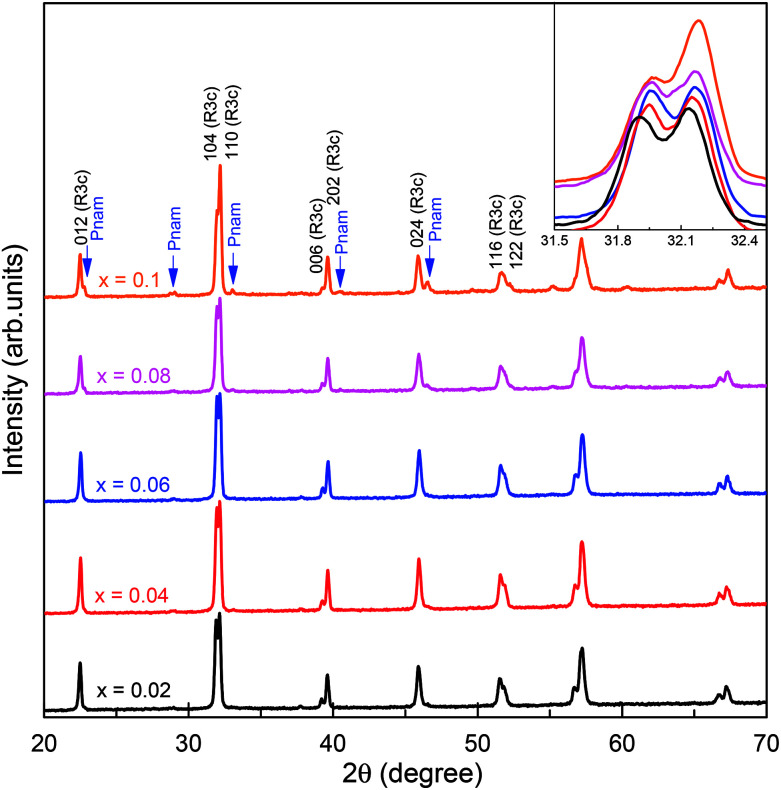
XRD patterns of Bi_0.88_Sm_0.12_Fe_1−*x*_Mn_*x*_O_3_ (BSFMO) samples. The inset shows the diffraction peaks at around 32°.

**Fig. 2:**
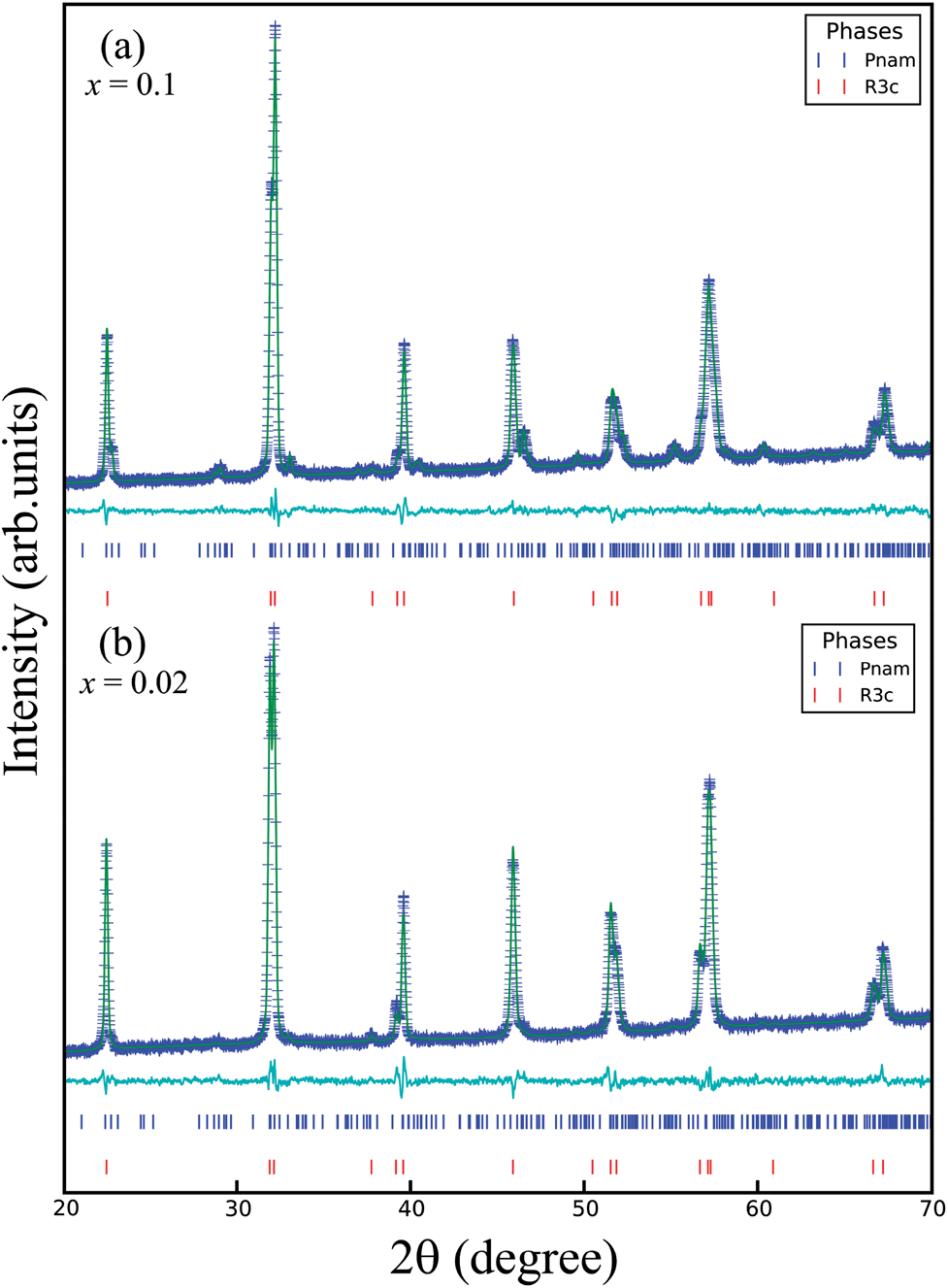
Rietveld refined XRD patterns of samples (a) *x* = 0.02 and (b) *x* = 0.1.

**Fig. 3:**
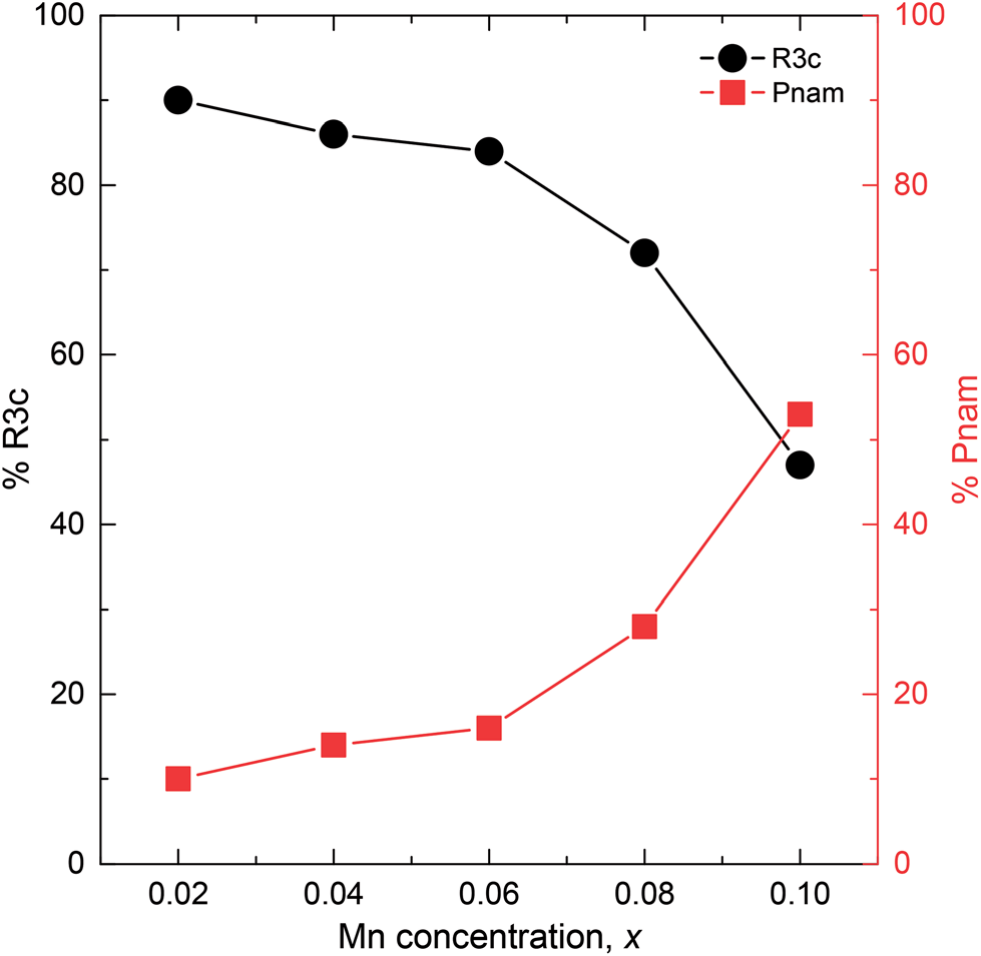
Variation of the crystalline phases as a function of Mn concentration.

Fig. 1 shows the XRD patterns of the BSFMO ceramics. All the main diffraction peaks of the BSFMO samples can be indexed on the basis of their rhombohedrally distorted perovskite structures with a space group *R*3*c* of BFO.^31^ No impurity phase was observed in the compounds within the detection limit of our XRD equipment. This suggests that the co-substitution of Sm and Mn at Bi and Fe sites can suppress the formation of the sillenite-type impurity phase such as Bi_25_FeO_4_ and Bi_2_Fe_4_O_9_, which is contrary to (La, Co),^19^ (Sm, Sc),^32^ and (Ce, Cr) codoped BFO.^33^ The inset of Fig. 1 reveals that the double diffraction peaks were located at around 32° shift slightly toward a high angle with an increasing Mn concentration, which indicated a small decrease in the lattice parameters. In addition, the inset of Fig. 1 shows a change in the relative intensity of the double diffraction peak, especially for the *x* = 0.1 sample. This can be attributed to an increase in the phase percentage of the antipolar *Pnam* orthorhombic structure and a decrease in the phase percentage of the polar *R*3*c* rhombohedral phase.^28^ The emergence of the antipolar phase is clearly observed in the XRD pattern of sample *x* = 0.1, for which all the diffraction peaks of the *Pnam* structure are well denoted in Fig. 1. Therefore, the substitution of Mn for Fe can induce a structural distortion in the *R*3*c* rhombohedral phase and drives a structural phase transition from a rhombohedral to an orthorhombic structure. The Mn-driven structural transition from a *R*3*c* rhombohedral to *Pnam* (isostructural with PbZrO_3_ perovskite) or *Imma* orthorhombic structure was previously observed with 15 wt% of Mn-doped Bi_0.9_Sm_0.1_FeO_3_.^28,34,35^ In the present work, we annealed samples at a higher temperature (930 °C) than that of ∼870 °C reported in the ref. 28 and 34. The structural transition could occur at the lower Mn concentration (below 10 wt% of Mn doping) because of the higher sintering temperature and Sm content in the BSFMO compounds. Our observation confirms that composition-driven structural transition is not only dependent on the dopant concentration but also on the sintering temperature. It is worth mentioning that a partial substitution of rare earth elements for BFO induced structural transformation from the ferroelectric phase to the antiferroelectric phase and then to a paraelectric phase.^36^ In general, the antiferroelectric phase had a PbZrO_3_-type orthorhombic structure, which can refer to either the *Pbam* or *Pnam* space groups. Thus, further increasing Mn concentration in BSFMO compounds may stabilize the antipolar phase and drive the phase transition to a nonpolar orthorhombic structure.^27,28^ To obtain more details on structural transformation, a Rietveld refinement of the XRD data was performed on the BSFMO samples by using simultaneous *R*3*c* rhombohedral and *Pnam* orthorhombic models with unit cells, respectively, 
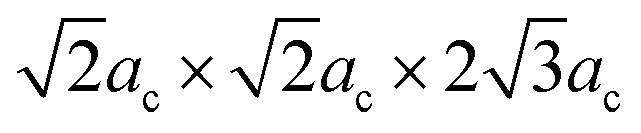
 and 
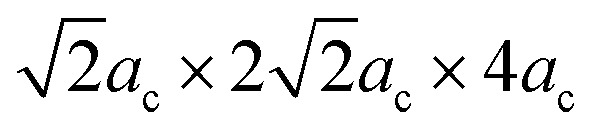
, where *a*_c_ ≈ 4 Å is the pseudocubic lattice parameter. The Rietveld-refined XRD patterns of the samples *x* = 0.02 and 0.1 are shown in Fig. 2. It is clear that all the diffraction peaks for two samples were well fitted to two-phase models, as indicated by the Bragg peaks (vertical bars) and the residue (solid lines). The lattice parameters and unit cell volumes of all the samples obtained from the Rietveld refinement are shown in Table 1. Initially, the *Pnam* phase percentage in the sample *x* = 0.02 was only 10%, which is very small compared to that of 90% in the *R*3*c* phase. With the increase of *x* from 0.02 to 0.1, the percentage of the *R*3*c* phase decreased continuously with an increase of the *Pnam* phase, as shown in Fig. 3. The percentage of the two phases was comparable in the sample *x* = 0.1. The evolution of the phase ratio and the shrink of the unit cell volume adequately support the above discussion on the shifting of the XRD peaks toward higher angles. The Rietveld refinement results confirmed that the BSFMO compounds show the MPB between the *R*3*c* rhombohedral and *Pnam* orthorhombic structures. The coexistence of the cycloidal and canted spin structures and their interaction at the PB would strongly affect the magnetic properties of BSFMO compounds.

**Table 1:** The crystal symmetry and lattice parameters of Bi_0.88_Sm_0.12_Fe_1−*x*_Mn_*x*_O_3_ compounds

Composition	Space group	*a* (Å)	*b* (Å)	*c* (Å)	*V* (Å)^3^
*x* = 0.02	*R*3*c* (90%)	5.5657(4)	5.5657(4)	13.7882(6)	369.89
	*Pnam* (10%)	5.6003(2)	11.2146(5)	15.6174(3)	980.85
*x* = 0.04	*R*3*c* (86%)	5.5643(7)	5.5643(7)	13.7795(9)	369.47
	*Pnam* (14%)	5.5864(3)	11.2497(6)	15.7249(8)	988.25
*x* = 0.06	*R*3*c* (84%)	5.5639(4)	5.5639(4)	13.7769(10)	369.35
	*Pnam* (16%)	5.5896(6)	11.2212(8)	15.6555(6)	981.93
*x* = 0.08	*R*3*c* (72%)	5.5631(6)	5.5631(6)	13.7731(11)	369.15
	*Pnam* (28%)	5.5926(7)	11.2126(9)	15.6238(12)	979.73
*x* = 0.10	*R*3*c* (47%)	5.5623(2)	5.5623(2)	13.7718(4)	369.00
	*Pnam* (53%)	5.5879(3)	11.2136(1)	15.6148(7)	978.443

**Fig. 4:**
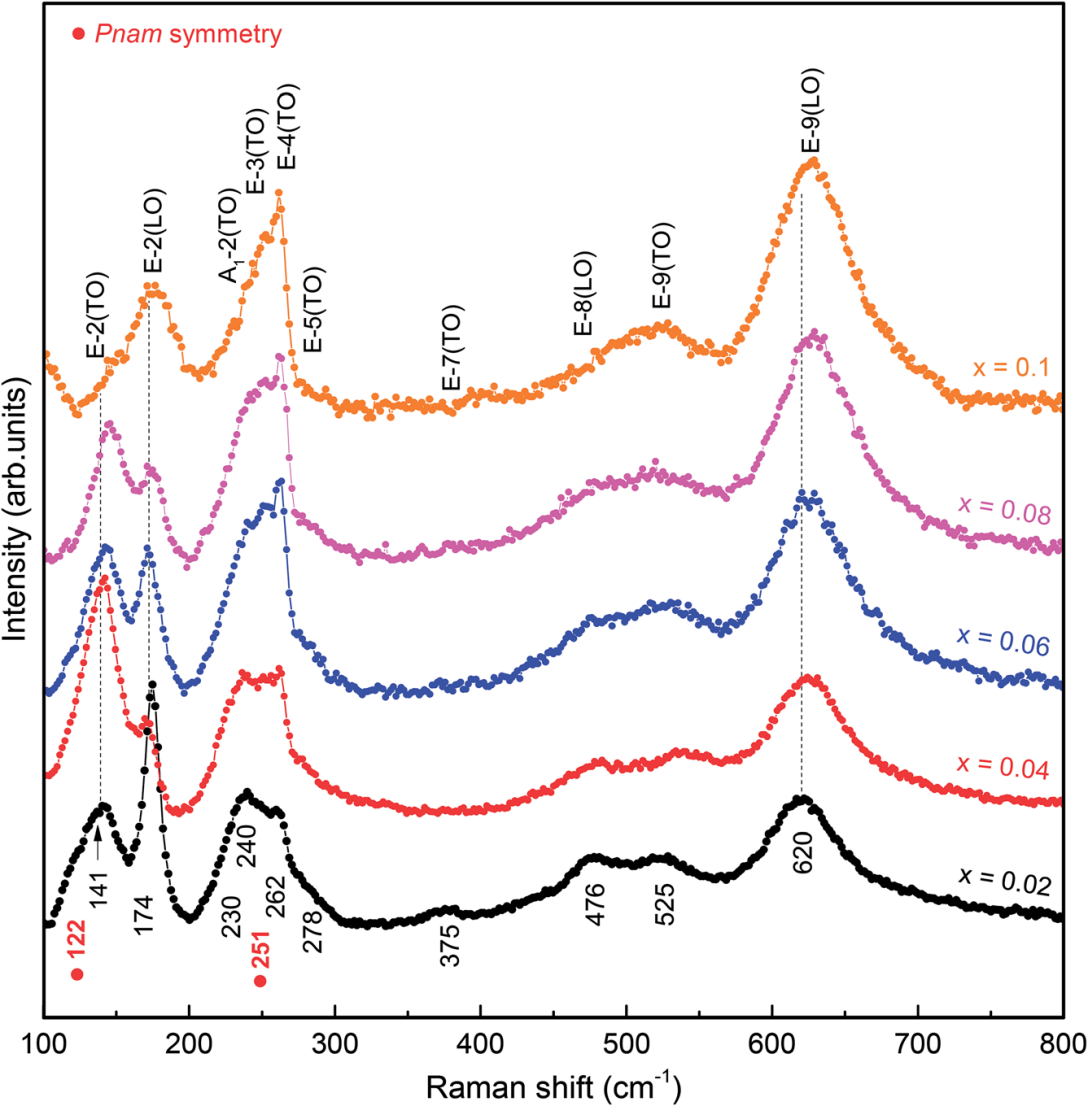
Raman scattering (RS) spectra of BSFMO samples.

**Fig. 5:**
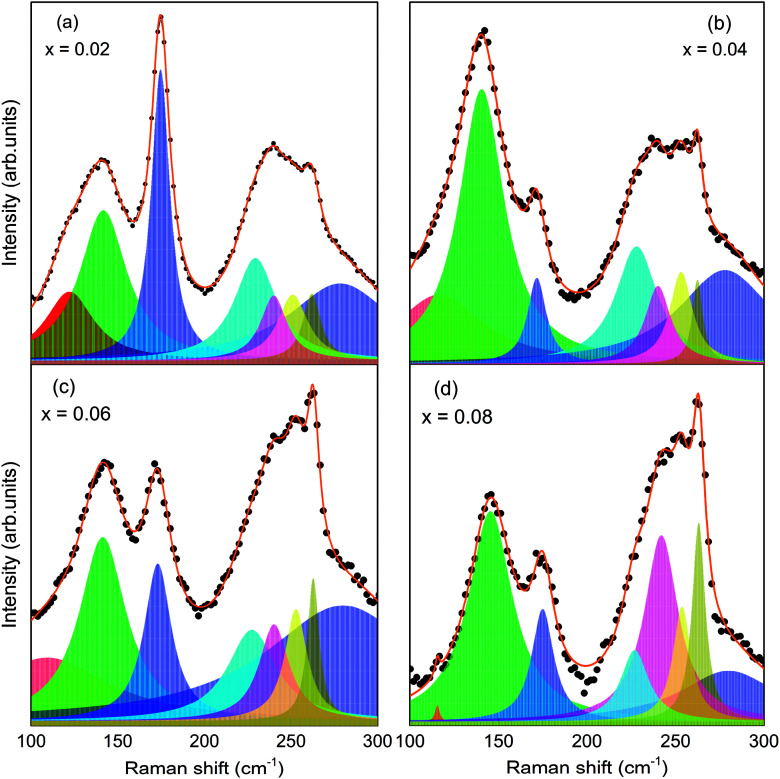
Typical deconvoluted Raman spectra for (a) *x* = 0.02, (b) *x* = 0.04, (c) *x* = 0.06, and (d) *x* = 0.08 in the region of 100–300 cm^−1^.

**Fig. 6:**
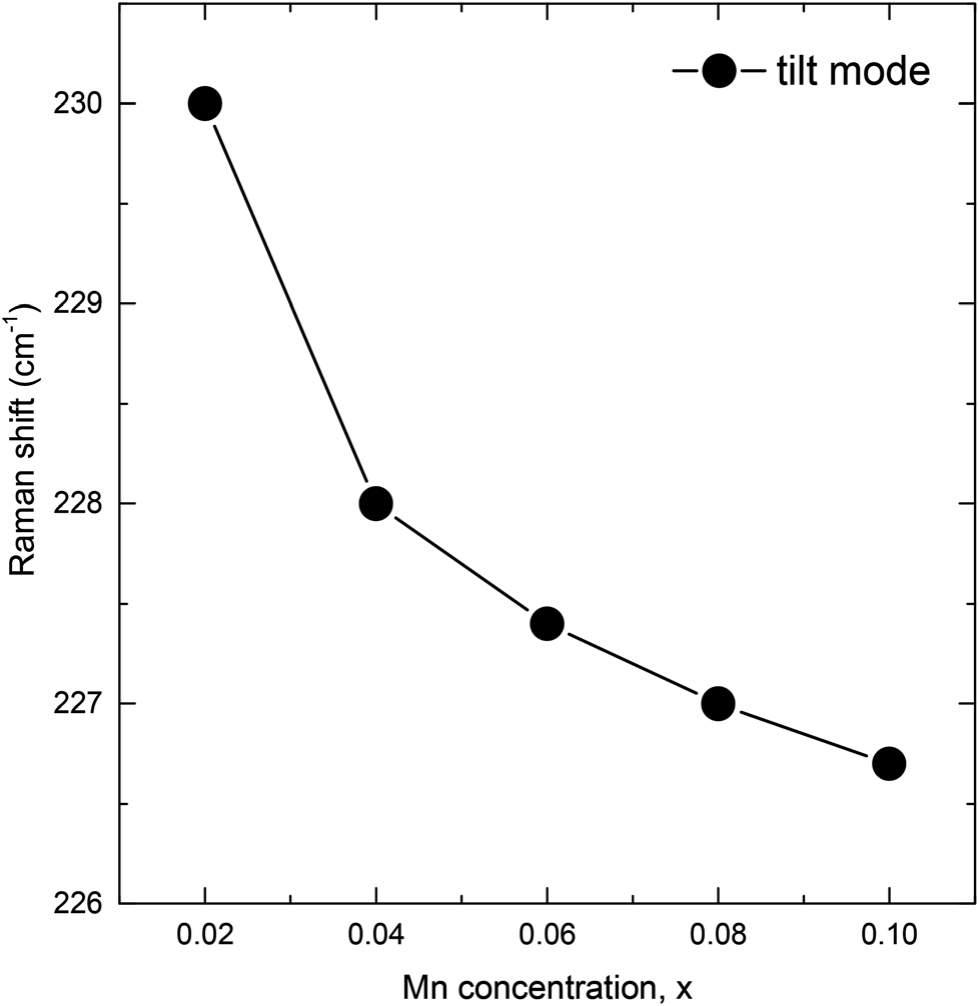
Position of the A_1_-2(TO) tilt mode as a function of Mn concentration.

Raman scattering (RS) is effective in studying the influence of a substitution on the crystal structure of polycrystalline compounds. In general, structural distortion and structural transition can be evaluated by the change of peak position, intensity, and bandwidth of the Raman modes. However, only a few attempts have been made with RS measurements to reveal a correlation between the crystal structure and physical properties of Sm and Mn codoped BFO.^24,25^ On the basis of the group theory, BFO has 13 Raman active modes within *R*3*c* rhombohedral symmetry. The vibration of the Bi lone pair contributes to the low frequency modes up to 167 cm^−1^, while Fe atoms are involved in the modes between 152 and 262 cm^−1^.^37^ Furthermore, structural distortion away from the initial *R*3*c* rhombohedral structure of BFO can be estimated by the change in intensity and frequency of the two characteristic modes at around 175 and 220 cm^−1^.^35^ In the present work, we measured the RS spectra at a wave number ranging from 100 to 800 cm^−1^. The RS spectra of the BSFMO samples and the deconvoluted RS spectra in a range of 100–300 cm^−1^ for the *x* = 0.02–0.08 samples are respectively shown in Fig. 4 and 5. All the samples displayed 12 Raman active modes, which were located at around 122, 141, 174, 230, 240, 251, 262, 278, 375, 476, 525, and 620 cm^−1^. Except for the modes at 122 and 251 cm^−1^, the Raman modes of the BSFMO compounds were in good agreement with the phonon vibration of the *R*3*c* rhombohedral symmetry of BFO.^38^ Two phonon modes at around 122 and 251 cm^−1^ should be related to the phonon vibration of the *Pnam* orthorhombic symmetry, because no impurity phase was detected in the BSFMO compounds.^19,39^ Our observations are consistent with previous reports.^19,40^ Therefore, these active modes of *R*3*c* symmetry can be assigned to the E-2(TO), E-2(LO), A_1_-2(TO), E-3(TO), E-4(TO), E-5(TO), E-7(TO), E-8(LO), E-9(TO), and E-9(LO), as denoted in Fig. 4.^35,38^ The high-frequency shift of E-2(TO) and E-2(LO) are attributed to the decrease of the Bi–O bond length, thus revealing shrinkage of unit cell volume. The Raman results are very consistent with the above discussion of the XRD analysis. The phase transformation toward a PbZrO_3_-type structure (*Pnam* symmetry) is also confirmed by the observation of suppression in the intensity of E-2(TO) and the broadening of E-2(LO) modes with an increasing Mn concentration, which is strongly consistent with the previous work of Bielecki *et al.*^35^ We now focus on the change of frequency of the tilt mode A_1_-2(TO). It is well known that the tilt mode frequency depends on the oxygen octahedral tilt angle (rotation along [111] axis) in *R*3*c* symmetry. The spectra deconvolution in Fig. 5 shows that the tilt mode decreased gradually when the Mn concentration increased, as plotted in Fig. 6. This implies that the tilt angle and the deviation of Fe^3+^–O–Fe^3+^ bond angle from 180° decreased with *x*, indicating that the cycloidal spin structure of the *R*3*c* symmetry cannot be suppressed by (Sm, Mn) codoped BFO. The results of the analysis of the RS spectra are in agreement with the XRD results.

**Fig. 7:**
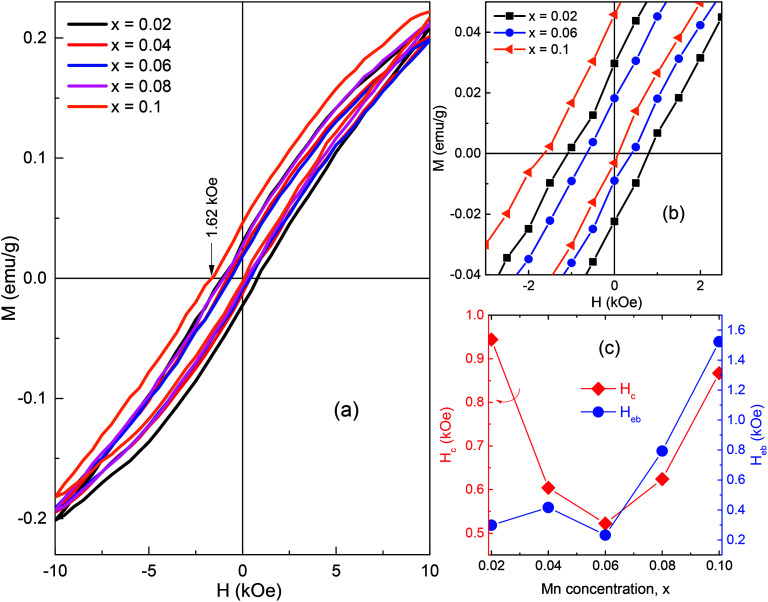
(a) *M*(*H*) loops of BSFMO samples measured immediately after synthesis. (b) The enlarged view around the origin of *x* = 0.02, 0.06, and 0.10 samples. (c) The *H*_c_ and *H*_eb_ as a function of Mn concentration.

The field dependence of magnetization obtained for the as-prepared BSFMO samples is shown in Fig. 7(a). The hysteresis loops of all the samples showed a small magnetization with a clear deviation from linearity, indicating the appearance of weak ferromagnetism (wFM) in Mn-doped Bi_0.88_Sm_0.12_FeO_3_ ceramic compounds. In general, the bulk BFO showed a linear magnetic field-dependent magnetization because the cycloidal modulation spin negated the macroscopic magnetization originating from a canting of the G-type antiferromagnetic (AFM) sublattices.^41^ The wFM observed in BSFMO polycrystalline ceramics stems from the suppression of the cycloidal spin order in the *R*3*c* and the collinear spin order in the *Pnam* phases. However, the analysis of the tilt mode frequency in the Raman spectra revealed that Mn substitution did not suppress the cycloid spin structure in the *R*3*c* symmetry. Instead, Mn doping can possibly increase the modulation periodicity of the cycloidal spin in BSFMO compounds, leading to a gradual degradation of remanent magnetization (*M*_r_) and coercive field (*H*_c_), as observed previously in Sm or Mn substitution for BFO.^42,43^ Indeed, the coercivity and remanent magnetization were continuously decreased in the samples *x* = 0.02–0.06, where the magnetic moments of the *R*3*c* mainly contribute to net magnetization as compared to the minority *Pnam* phase, as shown in Fig. 7(b) and (c). Because of remarkable increase of the *Pnam* phase percentage, samples *x* = 0.08 and 0.1 had high contribution of the *Pnam* phase to the net magnetic moments. A slight enhancement of the *M*_r_ and *H*_c_ were therefore observed in these samples. Our conclusions are in good agreement with the previous work of Khomchenko *et al.*^28^ Note that the *H*_c_ and *M*_r_ were respectively about 0.867 kOe and 0.023 emu g^−1^ for *x* = 0.1, which are still smaller than those of 0.944 kOe and 0.026 emu g^−1^ for *x* = 0.02. A further increase of the external field may have helped to obtain the higher coercivity and remanent magnetization contributed from the orthorhombic phase.^13^ The hysteresis loops of all the samples displayed the VHS effect. The VHS was found to increase after Mn substitution. This effect possibly derives from the inhomogeneous magnetic anisotropy of the two phases or magnetic coupling at the phase boundary, both of which were increased with an increase in the fraction of the *Pnam* phase at the expense of the *R*3*c* phase.^7,19^ The VHS can be estimated through the exchange bias field, *H*_eb_ = (*H*_c1_ + *H*_c2_)/2 (where *H*_c1_ and *H*_c2_ are the left and right coercive fields), as shown in Fig. 7(c).^16^ The *H*_eb_ value is rapidly increased from 0.2 kOe for *x* = 0.06 to 1.6 kOe for *x* = 0.1, corresponding to an increase in the *Pnam*/*R*3*c* phase ratio from 0.19 to 1.13.

**Fig. 8:**
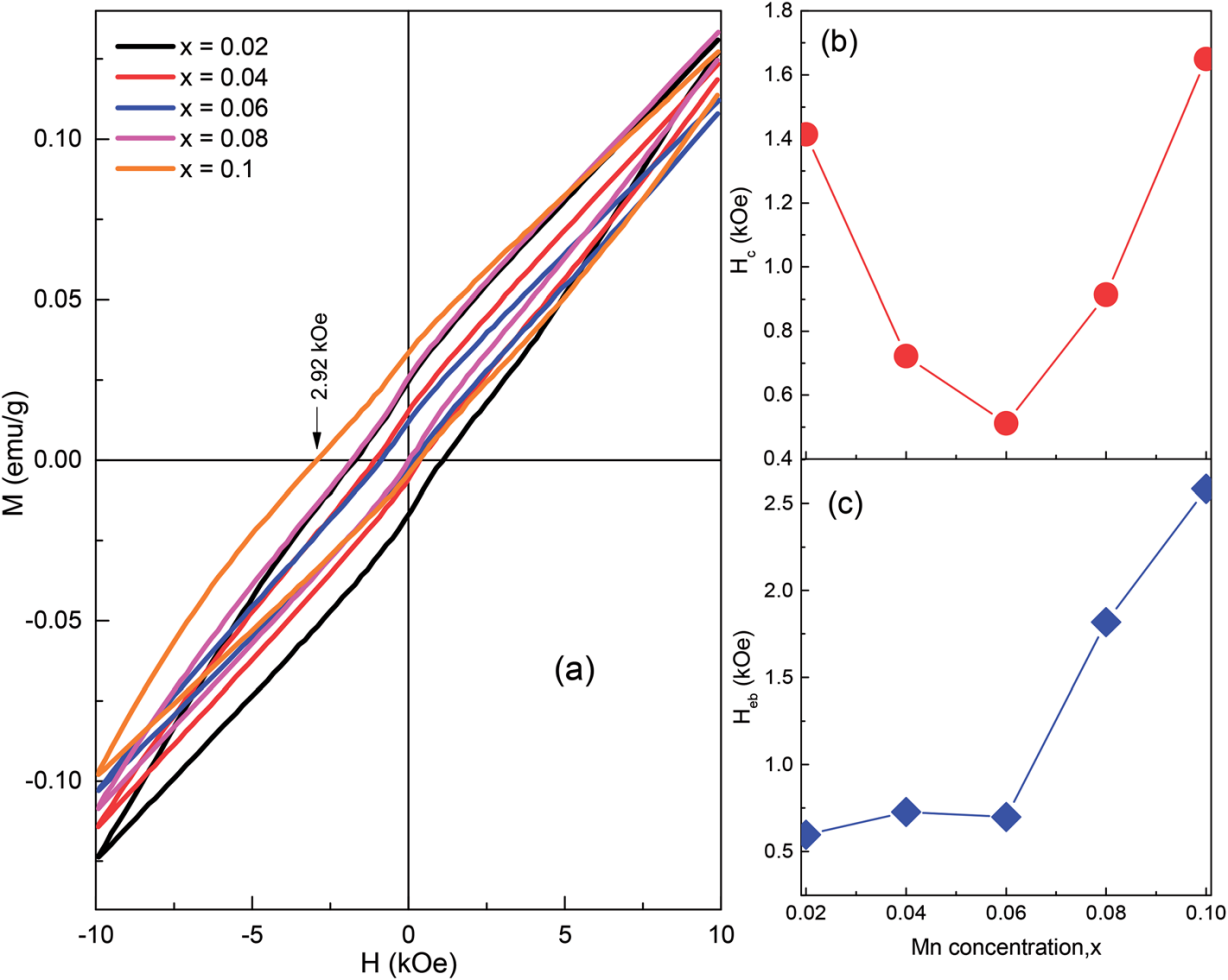
(a) Field dependence of magnetization obtained after 21 months. (b) and (c) The *H*_c_ and *H*_eb_ as a function of Mn concentration.

Fig. 8(a) shows the hysteresis loops of the BSFMO compounds obtained after 21 months of synthesis. The remanent magnetizations of all the samples were clearly reduced in comparison to that of the as-prepared samples. The *H*_c_ and *H*_eb_ values showed significant enhancement after 21 months of synthesis, as shown in Fig. 8(b) and (c). For instance, the *H*_c_ increased significantly from 0.944 kOe (as-prepared) to 1.415 kOe (after 21 months) for the sample *x* = 0.02 or from 0.867 kOe (as-prepared) to 1.649 kOe (after 21 months) for the sample *x* = 0.1. Thus, it is reasonable to conclude that the variation of the magnetic properties with time is related to the isothermal structural transition between the *R*3*c* and *Pnam* phases.^9,17^ Considering the self-phase transition from *R*3*c* to *Pnam*, the enhancement of coercivity is well understood because the collinear G-type AFM structure of the *Pnam* phase produces higher coercivity and remanent magnetization than that of the *R*3*c* phase. Indeed, the remanent magnetization and coercivity of a pure *Pnam* orthorhombic structure were respectively about ∼0.12 emu g^−1^ and ∼10 kOe.^22,28^ The reduction of remanent magnetization may be explained by the transition of *Pnam* to *R*3*c* phases. However, the self-phase transition was only observed when the rhombohedral transformed into the orthorhombic structure because of the metastable behavior of the rhombohedral structure at the MPB.^30,44,45^ Indeed, the XRD patterns obtained after 21 months of synthesis clearly confirm the self-phase transition from the *R*3*c* to *Pnam* phases (ESI†). Moreover, all the loops showed characteristics of the minor loop effect, which we are unable to compare to the saturation magnetization of the as-prepared and after 21 months samples. Furthermore, because of the higher magnetic anisotropy of the *Pnam* phase, a stronger external field would be required to saturate the magnetic moments. The isothermal structure transition is often observed in various compounds, but the study of the magnetic properties of the self-transformed structure is lacking.^46^ We have observed variation of magnetization with time in numerous compounds such as Zn-, Co-, or Ti-doped Bi_0.84_La_0.16_FeO_3_ at the MPB,^9,17,19^ and this behavior can only be explained through the self-transformation of crystal structural along with spin frustration at the PB.^19^ In the present work, the physical properties of the BSFMO compounds were stable for a longer time (21 months) than those of Bi_0.825_La_0.175_FeO_3_ ceramics (5 days)^44^ and Bi_0.84_La_0.16_Fe_1−*z*_M_*z*_O_3_ (2 months for M = Zn, 13 months for M = Ti, and 16 months for M = Co).^9,17,19^ As expected from the self-phase transition, the reconstruction of the spin structure should appear along with spin frustration at the PB, thereby forming PB ferromagnetism.^19^ The exchange anisotropy between the PB and the intrinsic AFM of the two phases reveals enhancement of the VHS, as shown in Fig. 8(c). It is worth mentioning that the spin frustration at the interface of the two structures is similar to the magnetic frustration on the AFM surface spin of the AFM/ferromagnetic bilayers, which possibly contributes to the enhancement of the coercivity and VHS effect.^47,48^

**Fig. 9:**
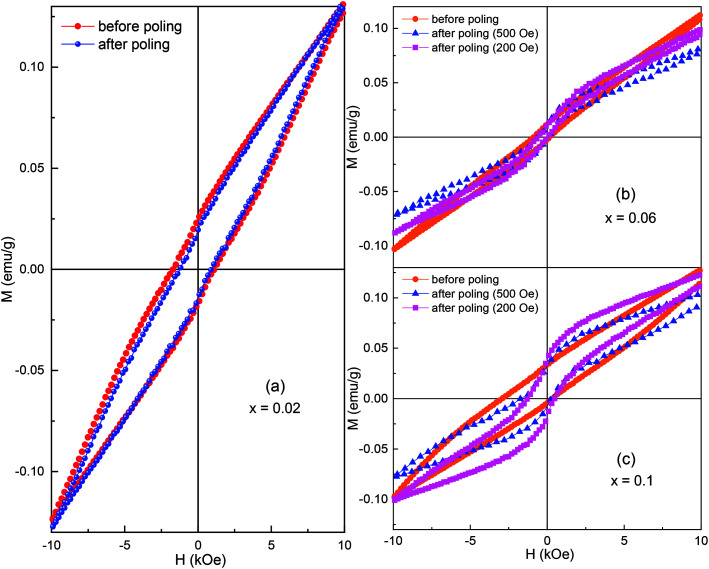
Room-temperature *M*–*H* loops of samples: (a) *x* = 0.02, (b) *x* = 0.06, and (c) *x* = 0.10 before and after poling in an electric field.

It is known that an electric field can induce irreversible and/or reversible phase transitions between antipolar orthorhombic and polar rhombohedral structures.^1,3,13,49,50^ Therefore, the electric field-induced phase transformation can possibly lead a disorder of spin at the PB and leaves evidence in the magnetic hysteresis loop. And hence, in the present work, we measured the magnetic hysteresis loops before and after applying an electric field of 17 kV cm^−1^ on the samples *x* = 0.02, 0.06, and 0.1, as shown in Fig. 9. If the switching of orthorhombic/rhombohedral phases is presented, the spin frustration possibly occurred at the PB and tailored the field step-dependent hysteresis loop and/or the ferromagnetic-like hysteresis loop.^6,9,19^ The sample *x* = 0.02 showed a small change of coercivity and remanent magnetization, while the other samples exhibited a significant change of coercivity and remanent magnetization. Obviously, the change of magnetic properties should be related to the electric field-induced phase switching between an orthorhombic and rhombohedral structure (ESI†). These observations confirm that the electric field control of magnetism is strongly dependent on the phase ratio. A remarkable change of magnetic properties was observed in the sample *x* = 0.1, where the *R*3*c* phase percentage was equal to the *Pnam* phase. The sample *x* = 0.02 had a large difference in the phase percentage that only showed a small change of coercivity and remanent magnetization. Interestingly, the samples *x* = 0.06 and 0.1 revealed a ferromagnetic-like hysteresis loop, confirming the coexistence of ferromagnetic and antiferromagnetic orders after applying the electric field.^7^ However, both the crystal structures have intrinsic antiferromagnetic behaviors, and the appearance of the ferromagnetic order could have only originated from PB ferromagnetism.^7,9,19^ Electric field-induced ferromagnetism can only be fulfilled by considering the spin frustration at the PB during the orthorhombic/rhombohedral phases switching. The frustrated spins at the PB form the PB ferromagnetic moments (*M*_PB_), which could possibly exchange its magnetic anisotropy with the intrinsic AFM of the two phases (*M*_AFM_). When a magnetic field is applied, the magnetic moments in the PB quickly respond to the change in the magnetic field than the *M*_AFM_. Therefore, the net magnetization increases rapidly with a change of the external field at the low field region (around origin). Ferromagnetic-like hysteresis loops are therefore observed. Moreover, it is interesting to observe the field step-dependent hysteresis loop, as shown in Fig. 9(b) and (c). The dependence of coercivity and remanent magnetization on the field step confirms the presence of the frustrated spin at the PB and the magnetic coupling between *M*_PB_ and *M*_AFM_.^19^ At a low field step, the exchange anisotropy between the *M*_PB_ and *M*_AFM_ helps to enhance the magnetization. At a high field step, the magnetic moments in the PB quickly follow to the applied field; hence, the weakening interaction of the *M*_PB_ and *M*_AFM_ produces smaller magnetization, as clearly observed in Fig. 9. Therefore, we believe that the VHS observed in the BSFMO compounds was related to magnetic coupling at the phase boundary rather than the inhomogeneous magnetic phases.

## 4. Conclusions

In summary, the effect of Mn doping on the crystal structure and magnetic properties of Bi_0.88_Sm_0.12_FeO_3_ ceramic compounds was investigated. The substitution of Mn for Fe can induce structural distortion in the *R*3*c* rhombohedral phase and drives a phase transition from *R*3*c* rhombohedral to a *Pnam* orthorhombic structure. The weak ferromagnetism observed in the ceramic compounds is mainly related to the *Pnam* phase, while the cycloidal spin structure in the rhombohedral phase cannot be suppressed. Studies on the self-phase transition and field-induced structural transition show a change of magnetization, which can be understood through the phase transition of the *R*3*c*-to-*Pnam* symmetries. The ferromagnetic-like hysteresis loops and field step-dependent hysteresis loop are fully explained by the frustrated spin at the PB and the magnetic coupling between *M*_PB_ and *M*_AFM_.

## Conflicts of interest

There are no conflicts to declare.

## Supplementary Material

RA-010-D0RA01642J-s001
