# Supplementary material for: Structural transition and magnetic properties of Mn doped Bi0.88Sm0.12FeO3 ceramics
Source: RSC Adv. 2020 Mar 26;10(20):11957–65. doi: 10.1039/d0ra01642j (PMC9050850; doi:10.1039/d0ra01642j)
Supplement: RA-010-D0RA01642J-s001 [file RA-010-D0RA01642J-s001.pdf]

Electronic Supplementary Material (ESI) for RSC Advances.
This journal is © The Royal Society of Chemistry 2020

Supplementary materials

Structural transition and magnetic properties of Mn doped $Bi_{0.88}Sm_{0.12}FeO_3$ ceramics

N. T. Hien[1,2], N. D. Vinh[3], N. V. Dang[3], T. T. Trang[3], H. T. Van[4], T. T. Thao[3], L. T. Hue[3], and P.T. Tho[3,*]

[1]Ceramics and Biomaterials Research Group, Advanced Institute of Materials Science, Ton Duc Thang University, Ho Chi Minh City, Vietnam
[2]Faculty of Applied Sciences, Ton Duc Thang University, Ho Chi Minh City, Vietnam
[3]Department of Physics and Chemistry, Thai Nguyen University of Sciences, Thai Nguyen, Vietnam
[4]Institute of Research and Development, Duy Tan University, Da Nang 550000, Vietnam

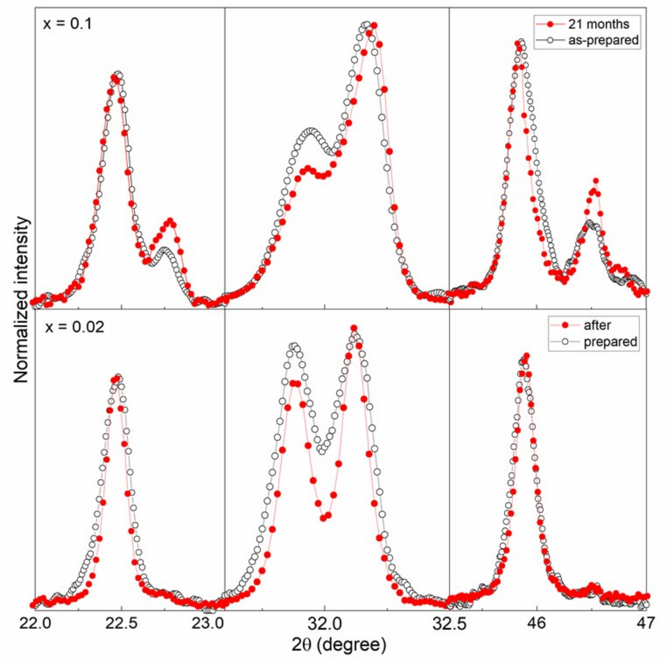

Figure S1. The comparison of XRD patterns of the as-prepared and after 21 months synthesis for $x = 0.02$ and 0.1 samples.

The change in relative intensity of the peaks at 2θ arounds 22.5, 32, and 46 confirm the isothermal structural transition from the *R3c* to *Pnam* phases.

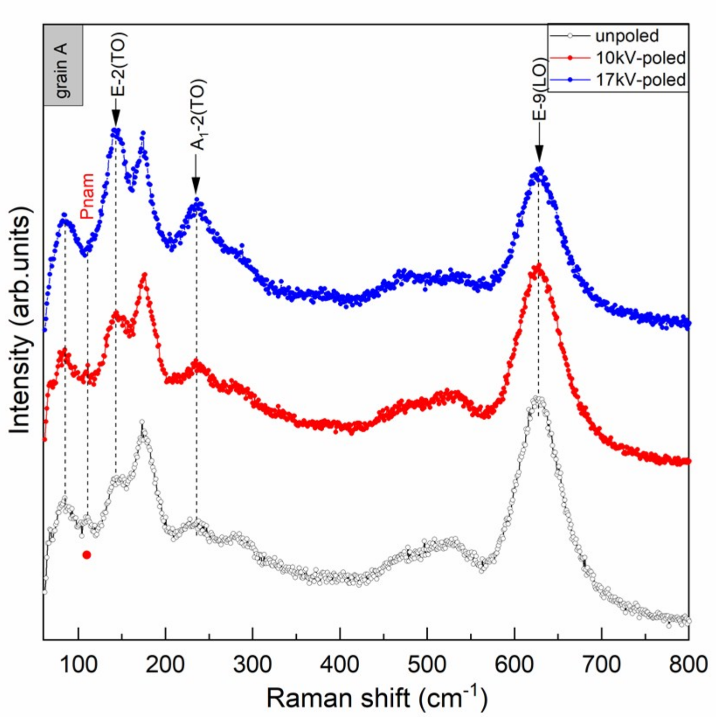

Figure S2. Raman spectra of sample x = 0.1 measured for single grain (grain-A) before and after poled in an electric field of 10 kV and 17 kV.

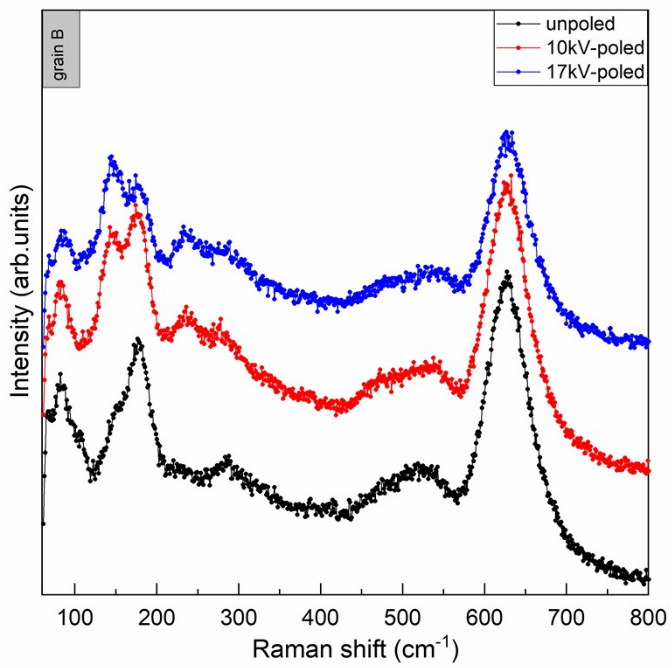

Figure S3. Raman spectra of sample x = 0.1 measured for single grain (grain-B) before and after poled in an electric field of 10 kV and 17 kV.

Figure S2, S3 shows the Raman spectra of sample $x = 0.1$ measured by Xpolar Raman 532 nm for different grain (grain A and B) before and after poled in an electric field of 10 kV and 17 kV. The E-2(TO), A_1-2(TO), and E-9(LO) modes are obviously changed their intensity in the grain A and B. The change in intensity of these modes are well confirmed the orthorhombic/rhombohedral phases switching.
